# Toward Development of a Vocal Fold Contact Pressure Probe: Sensor Characterization and Validation Using Synthetic Vocal Fold Models

**DOI:** 10.3390/app9153002

**Published:** 2019-07-26

**Authors:** Mohsen Motie-Shirazi, Matías Zañartu, Sean D. Peterson, Daryush D. Mehta, James B. Kobler, Robert E. Hillman, Byron D. Erath

**Affiliations:** 1 Department of Mechanical & Aeronautical Engineering, Clarkson University, Potsdam, NY 13699, USA; 2 Department of Electronic Engineering, Universidad Técnica Federico Santa María, Valparaíso 2390123, Chile; 3 Department of Mechanical and Mechatronics Engineering, University of Waterloo, Waterloo, ON N2L 3G1, Canada; 4 Center for Laryngeal Surgery and Voice Rehabilitation, Massachusetts General Hospital, Boston, MA 02114, USA

**Keywords:** vocal fold contact pressure, vocal fold collision

## Abstract

Excessive vocal fold collision pressures during phonation are considered to play a primary role in the formation of benign vocal fold lesions, such as nodules. The ability to accurately and reliably acquire intraglottal pressure has the potential to provide unique insights into the pathophysiology of phonotrauma. Difficulties arise, however, in directly measuring vocal fold contact pressures due to physical intrusion from the sensor that may disrupt the contact mechanics, as well as difficulty in determining probe/sensor position relative to the contact location. These issues are quantified and addressed through the implementation of a novel approach for identifying the timing and location of vocal fold contact, and measuring intraglottal and vocal fold contact pressures via a pressure probe embedded in the wall of a hemi-laryngeal flow facility. The accuracy and sensitivity of the pressure measurements are validated against ground truth values. Application to *in vivo* approaches are assessed by acquiring intraglottal and VF contact pressures using a synthetic, self-oscillating vocal fold model in a hemi-laryngeal configuration, where the sensitivity of the measured intraglottal and vocal fold contact pressure relative to the sensor position is explored.

## 1. Introduction

Voiced speech is produced as the vocal folds (VFs) undergo periodic oscillations arising from fluid-structure-acoustic [1–4] interactions. Each phonatory cycle is characterized by the opening and closing of the glottal area between the VFs [5], where voice quality is often dependent upon the degree of glottal closure [6]. Excessive contact forces that occur during glottal closure may, however, give rise to phonotrauma, which can be acute, or chronic [7]. Chronic behaviors can lead to phonotraumatic vocal hyperfunction [8], where compensation for a pathology, such as nodules, further exacerbates the underlying problem leading to a deleterious cycle of continual and increasing vocal fold tissue damage. Understanding physiological variables that influence VF contact forces can provide unique insights into the diagnosis, treatment, and remediation of voice disorders associated with phonotraumatic vocal hyperfunction.

Both direct and indirect measures of contact forces have been investigated using theoretical [9,10], experimental [11–14], and computational approaches [15–21]. Efforts focused on directly measuring VF contact are of particular interest as successful techniques would be easily applicable to clinical investigations.

**Figure 1.**
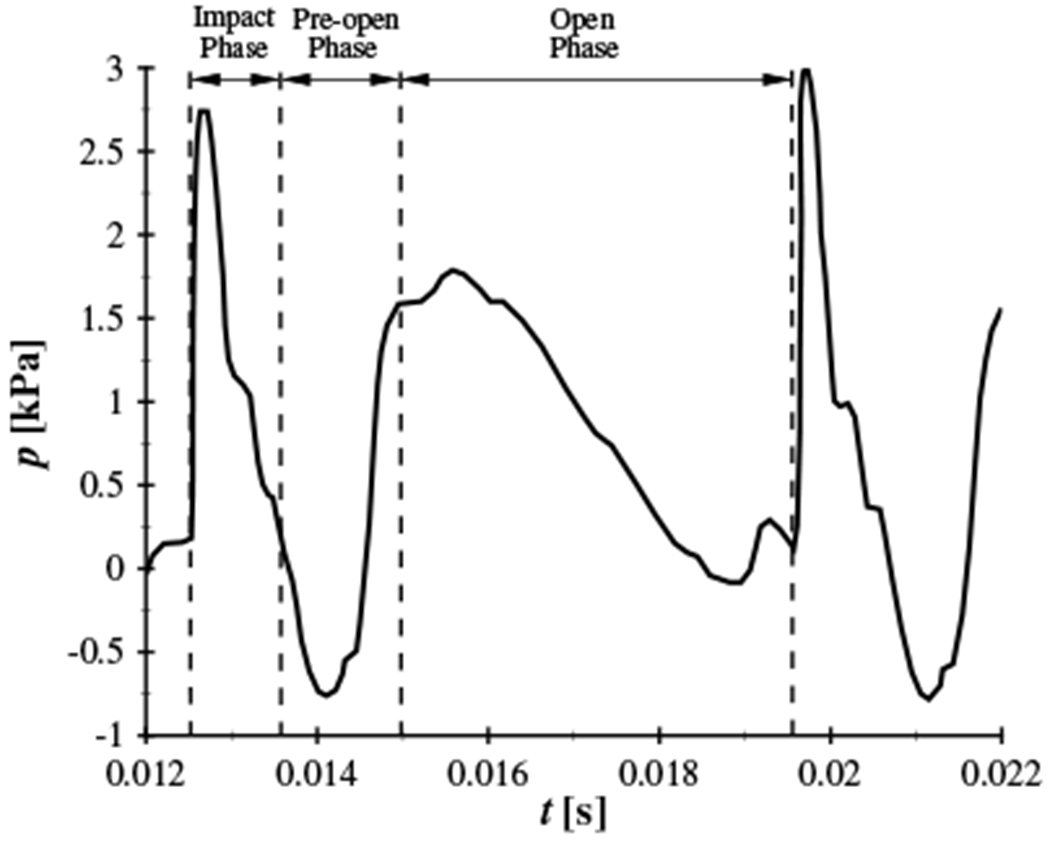
Intraglottal pressure recorded with a wall mounted pressure transducer in a self-oscillating, canine hemilarynx with a subglottal pressure of 1.96 kPa. Adapted from [11].

A representative intraglottal pressure waveform from early efforts investigating contact pressure in excised canine larnynges is presented in Figure 1 [11], where two clear peaks were identified in the time history of the pressure, with the primary peak arising from VF contact. As the VFs began to open in the Pre-Open Phase the wall-mounted pressure sensor was exposed to the subglottal pressure while the VFs were still closed, leading to the pressure rising before reaching a secondary peak during the Open Phase. The magnitude of the maximum contact pressure was correlated with the subglottal pressure, reaching values that were as high, or higher, than the subglottal pressure [11]. This cyclical, high amplitude, impact pressure is believed to produce cumulative damage in the VF structure, as most trauma becomes evident after prolonged voice usage [22–24].

Direct *in vivo* contact pressure measurements have, however, remained largely elusive due to the practical difficulties associated with sensor size, placement, and accuracy. Challenges arise due to (1) physical intrusion of the sensor, (2) the size of the sensor relative to the contact area, which may bias the recorded values due to poor spatial resolution, and (3) accurate positioning of any sensing element within the region of VF contact, as the VF contact length in the inferior-superior direction is O(mm) as the VFs are vibrating at O(100Hz).

Jiang and Titze, (1994) [11] acknowledged that the amount the pressure transducer was recessed into, or protruded above, the surrounding surface against which the VFs impacted had a significant influence on the magnitude of the measured contact pressure. While they chose to position the sensor such that it protruded slightly (~ 0.5mm) above the contact wall, no validation with a ground-truth measure of pressure was performed. Similar challenges were observed in the work of Gunter et. al., (2005) [25], who designed a custom, thin-film piezoelectric force-sensing element. Unfortunately, the size (~10mm × 15mm and 0.29mm thick) was on order with the size of the glottal opening. Additional approaches have sought to combat this challenge through implementation of flush-mounted sensors with synthetic, silicone VF models oscillating in a hemilaryngeal orientation [14,26].

To increase the spatial resolution of contact pressure measurements, smaller sensors have been utilized [14,27,28]. The first *in vivo* VF contact pressure measurements were carried out using a 1.8mm diameter piezoresistive sensor mounted on a curved, plastic-insulated metal cannula [28]. While VF contact pressures were acquired, the investigators noted significant difficulties in acquiring repeatable measures due to the challenge of accurately positioning the small sensor at the point of vocal fold contact in the vertical (inferior-superior) direction. Chen and Mongeau (2011) [14] utilized a 0.64mm diameter capillary tube connected to a microphone, with a small 0.51mm hole in the end of the tube, which was visually positioned at the point of VF contact. The sensitivity of the intraglottal pressure waveform to the sensor position was not reported.

If the sensor is large relative to the contact area, or positioned improperly, the measured pressure may be biased due to exposure to static air pressure during contact. At the extreme, this influence can be observed for the thin-film probe measurements of Gunter et. al., (2005) [25], where in addition to measuring VF contact, the inferior-superior sensor length of 15mm also simultaneously recorded both the subglottal and supraglottal pressure loadings during contact. This resulted in a periodic, triangular wave that did not capture the temporal features associated with intraglottal pressure measurements. In the work of Jiang and Titze (1994) [11] the influence of the sensor position relative to the location of contact was not discussed, although the pressure transducer was reported to have a sensing diameter of ~3mm, with the length of contact in the inferior-superior direction assumed to be ~3mm as well. Similarly, Weiss et. al., (2013) [26] developed a small Electro-Mechanical-Film sensor that was placed on a printed circuit board, and flush mounted into a wall. Preliminary data from synthetic VF models reported a double-peak intraglottal pressure waveform, with decreasing amplitudes in the anterior-posterior direction. The time between the two peaks was very short in comparison to prior investigations, and the pressure did not drop as much between them. The inferior-superior length of the sensor was 3mm, which could be expected to be as long, or longer than, the length of VF contact in the inferior-superior direction, thereby potentially biasing the pressure waveform.

To facilitate a robust investigation of the accuracy and sensitivity of VF contact measurements, a repeatable, benchtop, testing environment is needed. Synthetic, self-oscillating VF models, first developed by Thomson et al. [29], are an attractive surrogate for replicating the physics of contact, while also providing a suitable platform for parametric investigations. These models have been used ubiquitously in voiced speech investigations, including investigations of contact mechanics [13,14,26]. Initially proposed as a single-layer model [29], subsequent developments have incorporated multi-layered approaches with varying geometries to better mimic the layered tissue properties of the VFs [30–34]. As such, they provide a suitable alternative for replicating the mechanics of VF contact.

The objective of this work is to characterize and validate a new approach for acquiring intraglottal and VF contact pressure measurements with application to the design of an *in vivo* contact pressure sensor. Two experimental approaches and corresponding results are discussed sequentially. First, in Section 2, a procedure for measuring contact pressure via a wall-mounted sensor is characterized and validated using a custom-fabricated test platform. The facility enables comparison of contact pressure between the wall-mounted sensor and a ground-truth measure of contact pressure as a function of sensor intrusion, and the stiffness of the material contacting it. Next, this validated procedure for measuring contact pressure is implemented into a physical, hemi-laryngeal flow facility in Section 3, and the sensitivity of sensor position relative to contact location is quantified. Implications of the results from both studies, particularly with respect to *in vivo* VF contact pressure probe design, are then discussed in Section 4, and Section 5 is left for the conclusions.

## 2. Influence of Sensor Intrusion on Vocal Fold Contact Pressure Accuracy

A procedure for recording contact pressures using a wall-mounted pressure sensor is investigated herein. Emphasis is placed on validating the pressure measurements recorded by a pressure sensor with a ground truth value of contact pressure. The accuracy of the prescribed approach, and the sensitivity of contact pressure measurements to sensor intrusion, are discussed.

### 2.1. Contact Pressure Transducer Specifications

**Figure 2.**
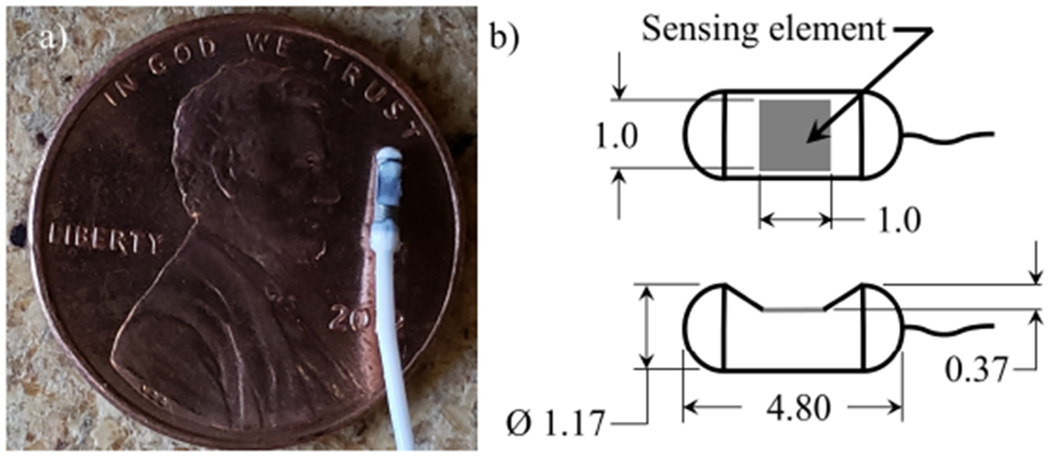
(**a**) Photograph of the Millar Mikro-Cath™ pressure transducer from a top view. (**b**) Schematic of the top and side views of the pressure transducer geometry and dimensions. Dimensions are shown in mm.

A Millar Mikro-Cath™ Pressure Catheter (Millar Inc., Houston, TX), designed to directly measure arterial pressure, was adapted for use as an *in vivo* contact pressure sensor. The transducer utilizes a piezoresistive sensing element to measure pressure over a dynamic range of −6.7 kPa to 40 kPa, and has a flat frequency response from DC to 10kHz, with an accuracy of ±1% of full-scale output (FSO) over the range of −6.7 kPa to 6.7 kPa. The size of the intraglottal pressure transducer is shown in Figure 2. The transducer is cylindrical in shape and has a diameter of 1.17mm, and is 4.80mm long. The sensing element that acquires the pressure is 1mm × 1mm in size, and is recessed from the upper surface of the cylindrical sensor by 0.37mm (see Figure 2). The sensor is powered by a two-channel signal conditioning unit (PCU-2000) that provides calibration and offset control. A 120cm long electrical cable connects the transducer to the signal conditioner.

Despite the small physical size of the intraglottal pressure transducer, it is expected to over-estimate impact pressure measurements because during collision, physical intrusion of the sensor causes local surface curvature of the contacting tissue. The stiffness of the contacting surface is also expected to influence the results, with stiffer surfaces resulting in decreased contact forces due to the tissue deforming less and not reaching the surface of the recessed sensing element.

### 2.2. Methods

**Figure 3.**
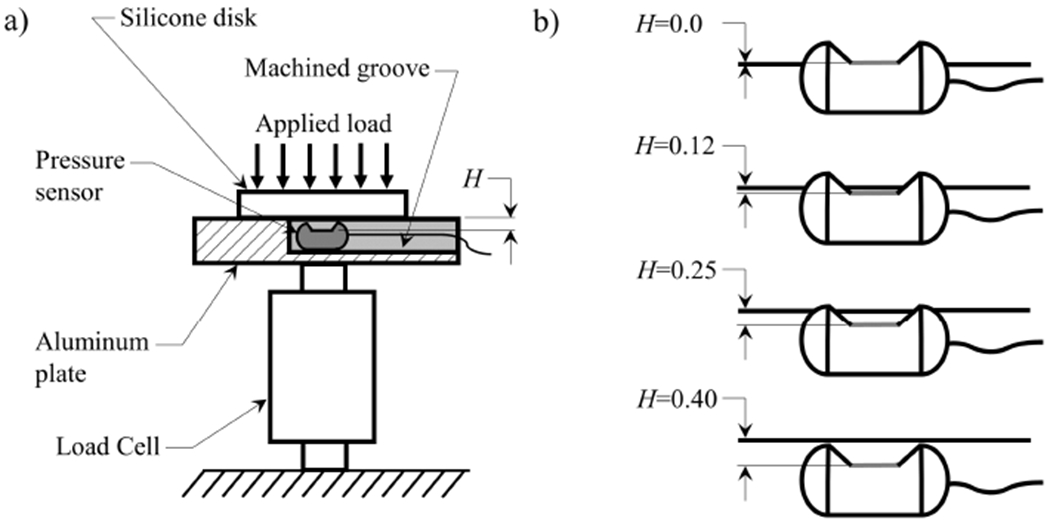
(**a**) Schematic of the custom facility for characterizing the pressure transducer performance in conditions representative of vocal fold contact. (**b**) Orientation of the pressure sensor relative to the surrounding contacting surface at the 4 sensor depths that were investigated. Dimensions are shown in mm.

To enable investigation of these behaviors, a custom facility, shown in Figure 3, was fabricated to enable characterization of the pressure transducer with a ground truth measure of contact pressure. The facility consisted of an Omega LCFD-1 Kg (2.2 lb) (Omega Engineering Inc., Norwalk, CT) force transducer, with ±0.15% FSO accuracy, which acted as a ground truth measure of static contact force. The force transducer has a 5.08cm diameter cylindrical aluminum plate affixed to it that acted as the primary contact surface. A silicone disk that measures 1.88cm in diameter and 0.94cm thick was fabricated from Smooth-On® Ecoflex 0030 (EF) (Smooth-On Inc., Macungie, PA, USA) silicone and placed on top of the aluminum plate. The elastic modulus of the silicone disk could be varied over a wide range based on the ratio of silicone to thinner that was used. The modulus of elasticity of each sample was measured using dynamic mechanical analysis on a TA Instruments Q800 at a frequency of 1 Hz and 5 % strain.

By placing the intraglottal pressure transducer between the aluminum plate and the silicone disk, and applying a uniformally-distributed static load to the silicone disk, the static pressure was acquired. The area of contact between the silicone disk and the aluminum plate was calculated by dyeing the surface of the silicone disk. For each discrete static loading that was applied to the silicone disk, the area of contact was obtained by measuring the diameter of the colored dye that was transferred to the aluminum plate during loading. The ground truth contact pressure was then computed as the output of the force sensor divided by the contact area of the silicone disk. Each measurement was repeated three times.

Individual aluminum contact plates were fabricated with different groove depths, such that the height of the transducer intrusion, *H*, (see Figure 3) could be varied. Specifically, *H* prescribes the distance between the surface of the sensing element of the pressure transducer and the surface of the aluminum plate against which the silicone disk is pressed.

### 2.3. Results

#### 2.3.1. Sensor Intrusion and Tissue Stiffness Dependency

**Figure 4.**
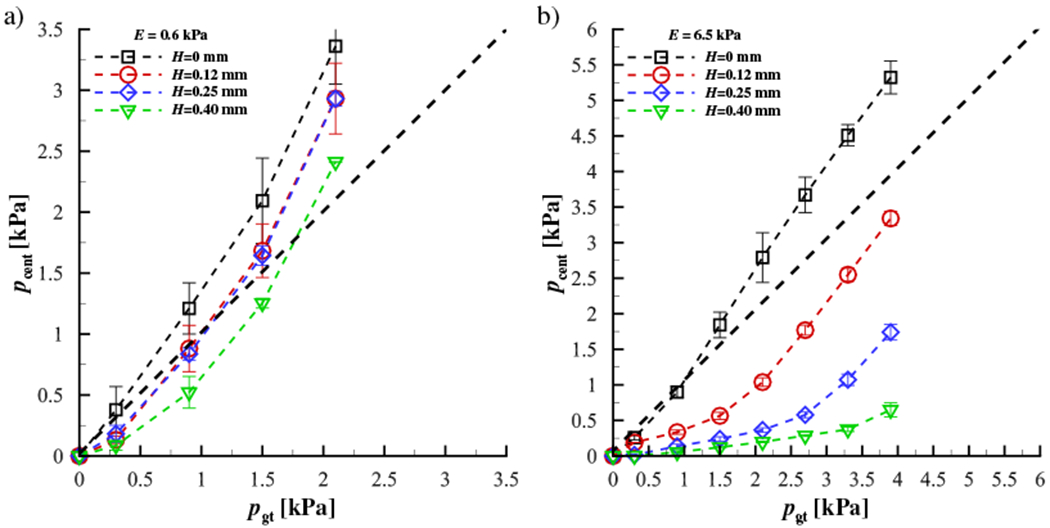
Variation of the pressure at the center of the contacting silicone disk as a function of offset height between the surface of the sensing element on the pressure transducer, and the surrounding contact surface of the aluminum plate (see Figure 2) for (**a**) a modulus of elasticity of *E* = 0.6 kPa for the contacting silicone disk, and (**b**) a modulus of elasticity of *E* = 6.5 kPa for the contacting silicone disk. In both plots, the line of one-to-one correspondence between the measured and ground truth pressure is denoted by the heavy, dashed, straight line. The symbols denote the mean value and the bars indicate the maximum and minimum values for each trial.

The results are plotted in Figure 4a,b for a contacting silicone disk with a modulus of elasticity of *E* = 0.6 kPa and *E* = 6.5 kPa, respectively. The range of elastic moduli was chosen to be representative of the VF cover layer [35,36]. In both plots, *p*_cent_ is the pressure measured by the pressure transducer at the centerpoint of the contacting silicone disk and *p*_gt_ is the ground truth pressure from the force transducer measurements. The mean measurement values are plotted as symbols, while the error bars indicate the extents of the maximum and minimum values for each set of measurements.The heavy solid dashed line indicate a one-to-one correspondence between the measured and ground truth pressures. As shown in Figure 4a, a strong dependency is observed based on the height of the surface of the sensing element relative to the surrounding contact surface. As expected, increasing sensor protrusion above the surrounding contact surface results in over-prediction of the contact pressure due to the physical deformation of the silicone disk as it contacts the pressure transducer, increasing the local stress. Figure 4b demonstrates that for a stiffer contacting material, the measured pressure decreases due to the material deforming less, and therefore not contacting the recessed surface of the sensing element. This becomes more pronounced as the surface of the sensing element becomes recessed below the surrounding contact surface (*H* ≥ 0.25mm). A sensor that protrudes or is recessed, even by values < 0.5mm relative to the surrounding contacting surface, can have a drastic influence on the contact pressure that is measured, with errors greater than 100%.

**Figure 5.**
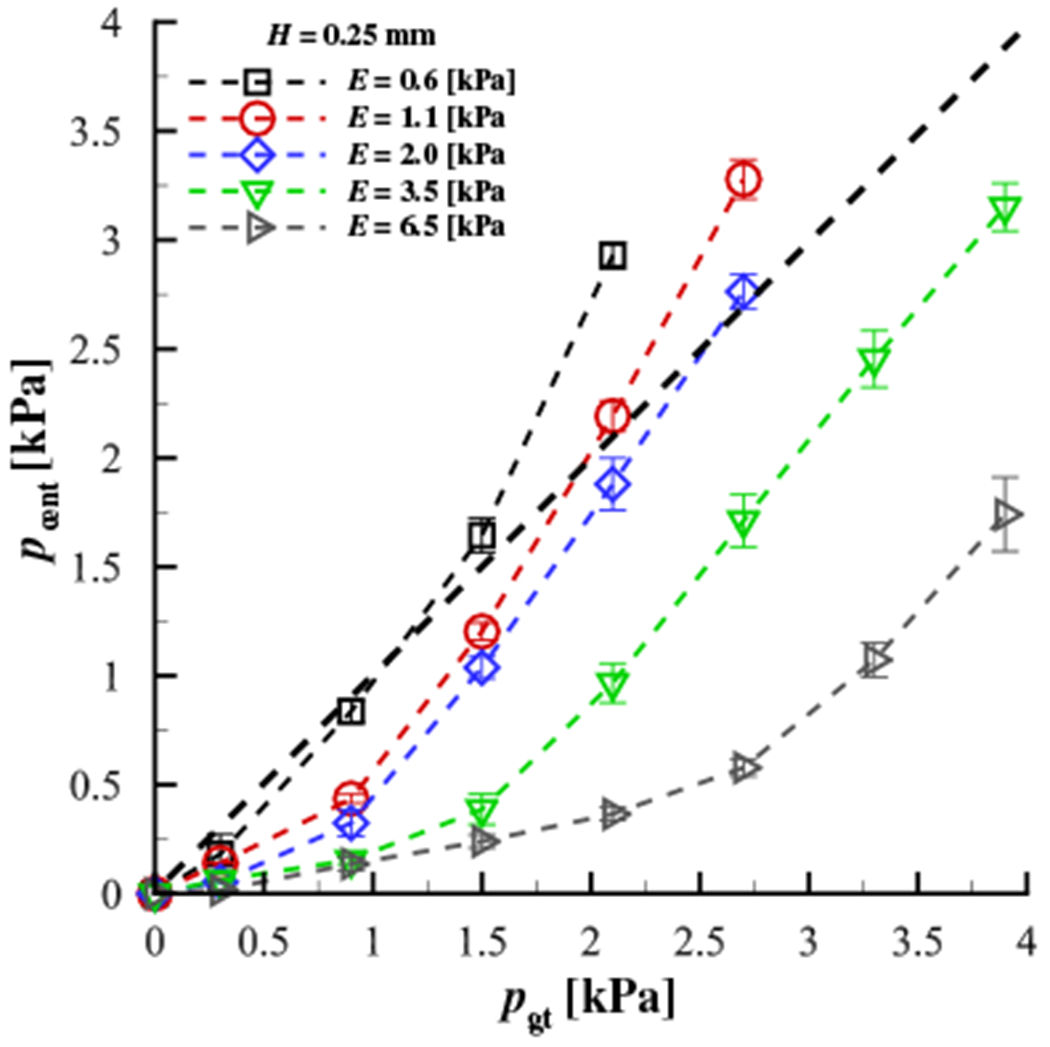
Variation of the pressure at the center of the contacting silicone disk as a function of the modulus of elasticity of the disk, for an offset distance of *H* = 0.25mm (see Figure 2). The line of one-to-one correspondence between the measured and ground truth pressure is denoted by the heavy, dashed, straight line. The symbols denote the mean value and the bars indicate the maximum and minimum values for each trial.

To quantify the dependency of the measured contact pressure as a function of the stiffness of the contacting material, Figure 5 presents the measured centerline contact pressure versus the ground truth pressure where the modulus of elasticity of the contacting silicone disk varies from *E* = 0.6 kPa to *E* = 6.5 kPa, while the height of the surface of the sensing element relative to the surrounding contact surface is maintained constant at *H* = 0.25mm. As the material stiffness increases the transducer begins to significantly under-predict the actual contact pressure. This is because the surface of the sensing element is recessed down into the transducer (see Figure 2), and as such, the amount that the material will deform around the outer surface of the transducer and impact the surface of the sensing element depends on the material stiffness. This is problematic for *in vivo* investigations where the tissue stiffness is not known *a-priori*. These measurements highlight the potential difficulties in acquiring direct measures of contact pressure with a physical probe, where intrusion of the sensor and variable stiffness of the contacting material can corrupt the measurement in complex, nonlinear ways.

#### 2.3.2. Mitigating Sensor Intrusion Using a Wall-Mounted, Embedded Approach

To address the aforementioned challenges, an approach to ensure one-to-one agreement between the pressure recorded by the Millar Mikro-Cath™ transducer and the ground truth pressure obtained from the force transducer was developed, whereby the pressure transducer is placed in a groove machined in the aluminum plate (see Figure 3) with the surface of the sensing element oriented upwards towards the contacting surface. The groove is then filled with Smooth-On® Dragon Skin 10 (DS) silicone and while it is still liquid the excess material is wiped from the surface of the aluminum plate with a razor blade to ensure the top surface of the silicone is perfectly flush with the surrounding plate. The silicone is then allowed to cure. This approach ensures a perfectly flat surface for contact, which avoids measurement artifacts due to physical interference caused by the sensor. Encapsulating the sensor in silicone does, however, allow the transmittal of the pressure applied at the surface of contact onto the sensing element.

**Figure 6.**
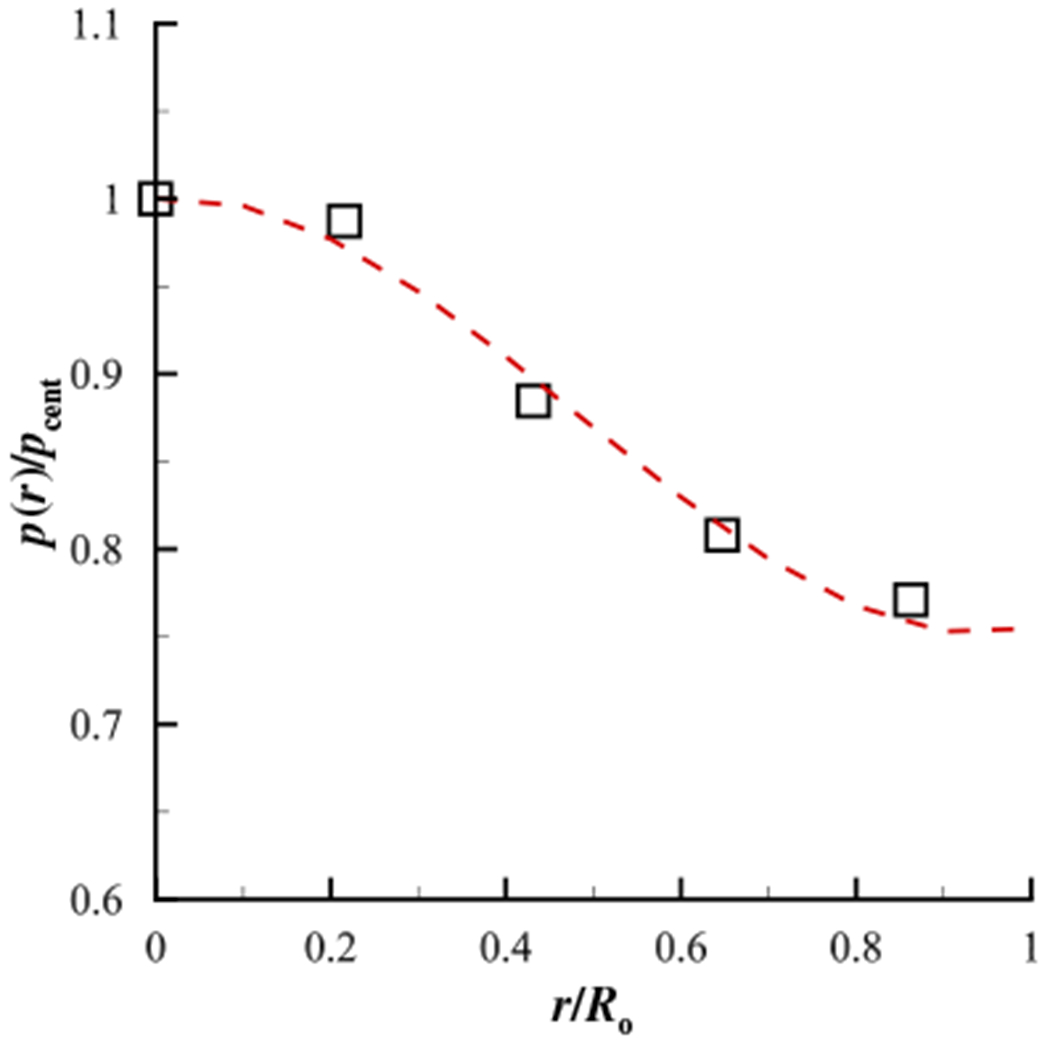
Distribution of the pressure for the *E* = 1.1 kPa silicone disk as a function of radial position with a 3^rd^-order polynomial fit overlaid. The polynomial fit is given by *p*(*r*)/*p*_cent_ = 0.6438(*r*/*R*_o_)^3^ − 0.9412(*r*/*R*_o_)^2^ + 0.04751(*r*/*R*_o_) + 1.0.

Due to the manner in which the silicone disk was loaded during testing, it was found that the pressure measured by the transducer varied based on whether it was at the center, or edge of the disk (i.e., the applied force was not uniform over the entire area of the disk). This variation influenced the computation of the ground truth pressure, which was computed as the output from the force transducer divided by the area of contact, which assumes a uniform pressure distribution under the contacting disk. To account for the non-uniform pressure distribution under the disk, the contact pressure was measured at 5 radial positions extending from the centerpoint to the edge of the disk. The radial position was normalized by the radius of the silicone disk, and the pressure was normalized by the pressure at the center. For each case, the distribution of the pressure was then plotted and a 3rd-order polynomial was fit to the data points, The polynomial fit was performed to enable easy computation of the spatially-averaged contact pressure (*p*_m,avg_) by integrating over the area of the silicone disk. This pressure was then compared with the ground-truth pressure values computed with the force sensor. An exemplary plot of the nondimensionalized pressure as a function of nondimensional radial position for a silicone disk with an elastic modulus of *E* = 1.1 kPa is shown in Figure 6, where the overlaid polynomial fit is expressed as *p*(*r*)/*p*_cent_ = 0.6438(*r*/*R*_o_)^3^ – 0.9412(*r*/*R*_o_)^2^ + 0.04751(*r*/*R*_o_) + 1.0, where r is the radial position measured from the center of the disk, and *R*_o_ is the outer radius of the silicone disk. For all investigated elastic moduli, the *R*^2^ value of the polynomial fit was > 0.996.

**Figure 7.**
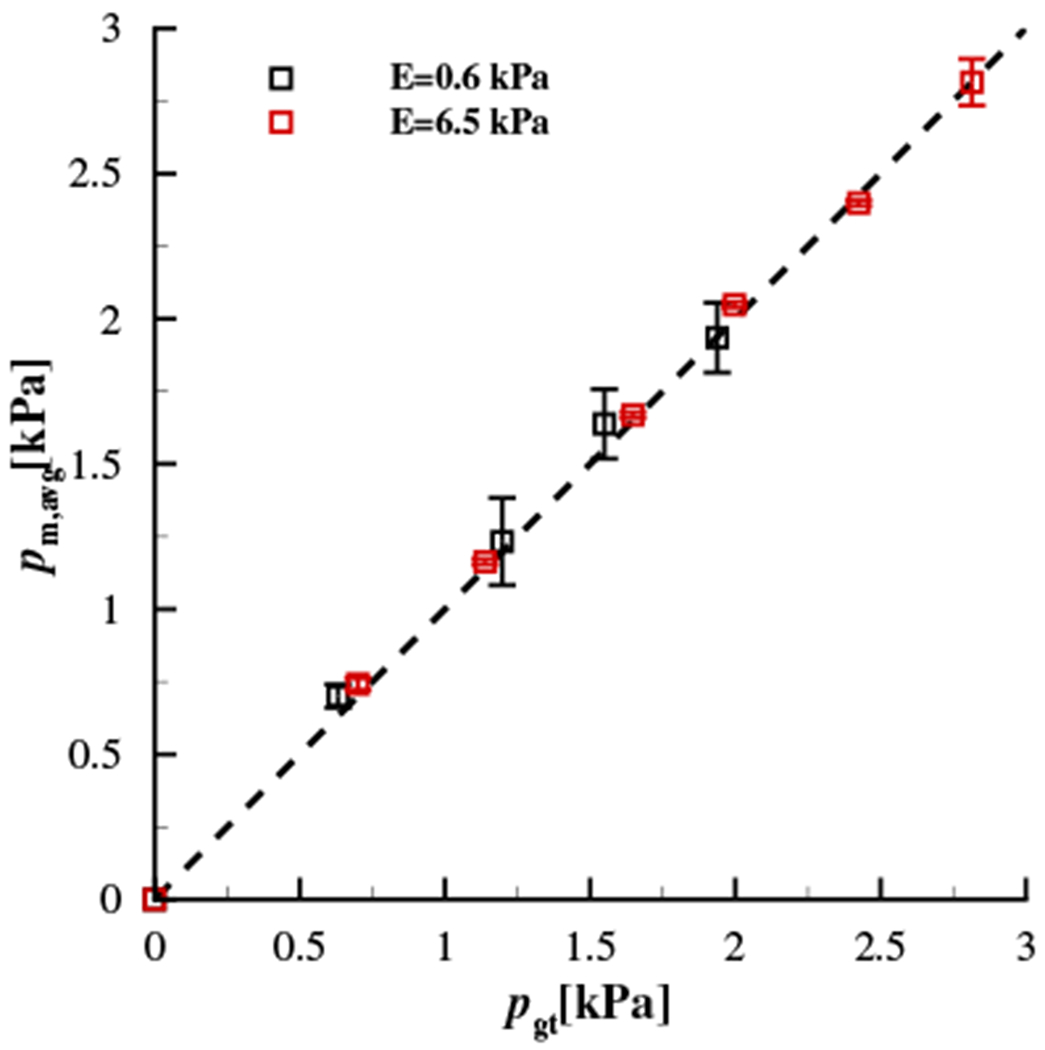
Validation of the embedded pressure transducer. The spatially-averaged pressure *p*_m,avg_ measured by the pressure transducer embedded in Smooth-On® DS silicone is plotted versus the ground truth pressure, *p*_gt_, computed from the force transducer and the contact area. Data are presented for elastic moduli of *E* = 0.6 kPa and *E* = 6.5 kPa for the contacting silicone disk. The heavy dashed line denotes one-to-one correspondence between the measured and ground truth pressures. Five measurements were performed at each point. The symbols denote the mean value and the bars indicate the maximum and minimum values for each trial.

In order to validate the approach of embedding the sensor in a silicone channel to acquire an accurate measure of the contact pressure, the pressure transducer was recalibrated by submerging it in varying depths of water, and comparing the hydrostatic pressure with that recorded by the pressure transducer. Comparisons between the spatially-averaged pressure loading over the contacting silicone disk recorded by the Millar Mikro-Cath™ pressure transducer and the ground truth measure from the force transducer were then acquired at various discrete, static loads, and as a function of the elastic modulus of the contacting material. Figure 7 shows the results at the limits for which the elastic modulus was investigated. For each load, five separate measurements were performed. The mean values are reported, with the maximum and minimum values for each trial represented by the bars. Note that very good agreement is found between the measured and ground-truth pressures, and it is independent of the elastic modulus of the contacting material, validating the approach for measuring contact pressure. Finally, the frequency response of the sensor embedded in the silicone-filled groove was found to be > 3.8kHz, which covers the majority of the bandwidth required to characterize voiced sound production.

## 3. Sensitivity of the Intraglottal and Vocal Fold Contact Pressures to Sensor Position

Following validation of the pressure sensor and the associated approach for measuring contact pressure on a wall, the following section discusses its implementation into a self-oscillating hemi-laryngeal VF flow facility and the investigation of the sensitivity of VF contact pressure measurements to pressure sensor location. The synthetic VF model utilized for the experiments is described in Section 3.1.1, while the specifics of the flow facility are discussed in Section 3.1.2. The synthetic VF model kinematics as a suitable surrogate for *in vivo* investigations are validated in Section 3.2.1. Finally, the intraglottal pressure acquisitions in the model laryngeal flow facility, and the sensitivity of transducer position relative to contact location are discussed in Section 3.2.2 and Section 3.2.3, respectively.

### 3.1. Methods

#### 3.1.1. Synthetic Vocal Fold Model Design

**Figure 8.**
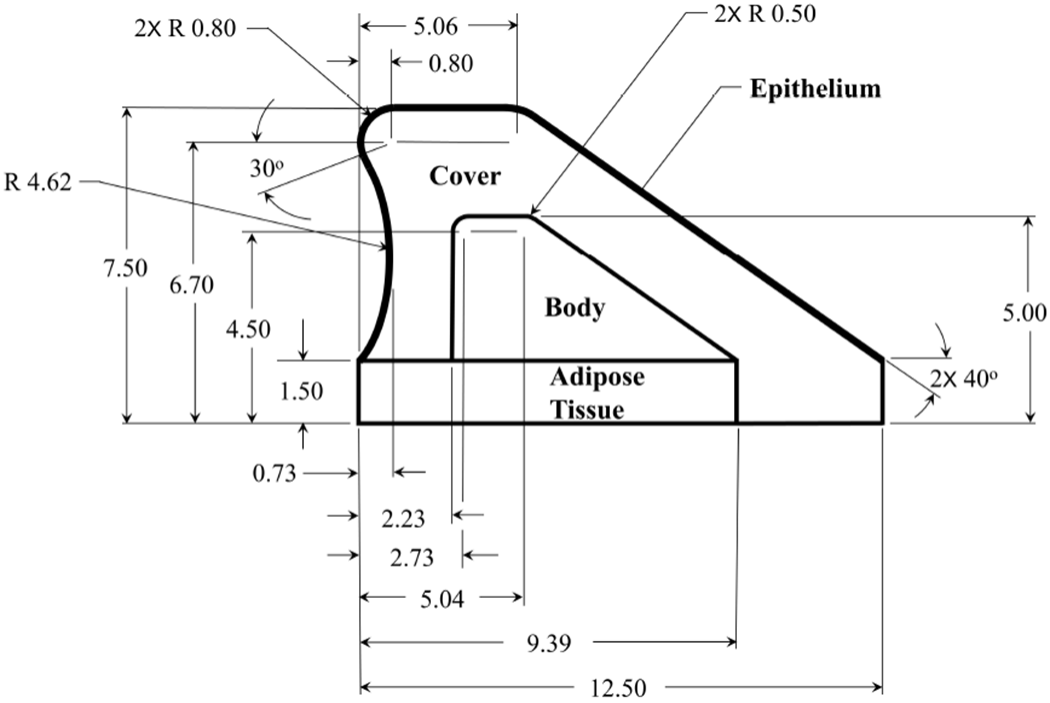
Geometry and dimensions of the layered synthetic vocal fold model. All dimensions are in mm.

All synthetic VF model contact investigations were performed using layered, silicone, self-oscillating models of the VFs, with differing moduli of elasticity in each layer. The approach employed herein follows prior work [29–34], albeit with some modifications to the geometry. The peripheral geometry of the VF model prescribed in the current work was developed based on both previously reported dimensions along a mid-membranous coronal section of the VFs [37], as well as visual observation of stained slices of the human VFs [38]. The resultant VF profile and associated dimensions are presented in Figure 8. The profile was extruded in the anterior-posterior direction to a length of 1.7cm. Two notable deviations from prior work include (1) an undercut along the superior surface that corresponds to the laryngeal ventricle and (2) addition of the paraglottic space [39], which comprises the space between the thyroarytenoid muscle and the thyroid cartilage, and was modeled as a substrate layer of adipose tissue (AT) (i.e., fatty tissue). Initial investigations, not shown here for brevity, demonstrated that the inclusion of an undercut produced a more pronounced convergent-divergent mucosal wave, and a longer inferior-superior contact length. Inclusion of the AT layer resulted in significantly lower VF modal frequencies [40]. The dimensions of the AT layer were estimated based on histological images of the larynx [38].

Each layer of the VF was manufactured using Smooth-On® silicone rubber. Both Ecoflex 0030 (EF) and Dragon Skin 10 (DS) were mixed with varying ratios of silicone thinner to create the desired modulus of elasticity. Characterization of the modulus of elasticity was performed via dynamic mechanical analysis on a TA Instruments Q800 at a frequency of 1Hz and 5% strain. Three samples were tested for each measurement of the modulus of elasticity. Table 1 presents: the layer of the VF model, the range of physiological values for the modulus of elasticity, the resultant modulus of elasticity of the current synthetic silicone VF model, and the ratio of part A and B silicone to thinner by weight, represented as a ratio of A:B:Thinner. The measured values are reported as the mean and the standard deviation for each layer. The models were cast and cured in successive layers, starting with the AT, and ending with the epithelium, similar to prior studies [33]. Note, the dimensions shown in Figure 8 are for the AT, Body, and Cover layers. The final epithelial layer was produced by pouring two separate layers of silicone rubber over the model, allowing each layer to cure before pouring the next. This increased the medial-lateral height of the model by ~ 0.25mm, resulting in a total VF height of ~7.75mm.

#### 3.1.2. Laryngeal Flow Facility

**Figure 9.**
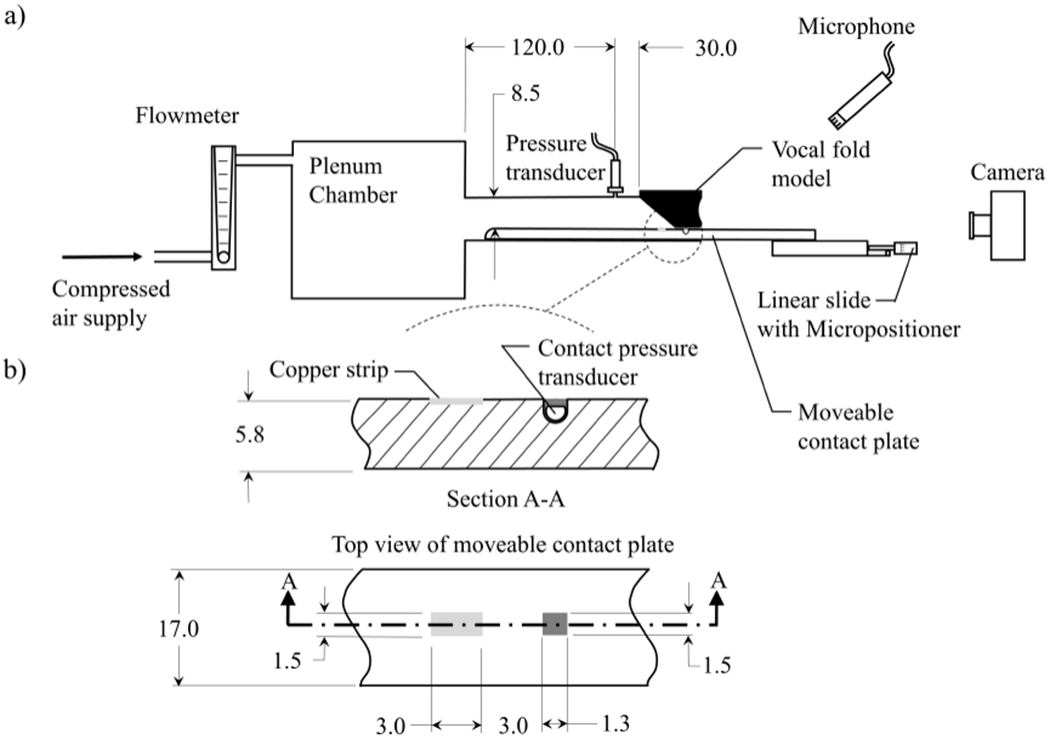
(**a**) Schematic of the experimental flow facility. (**b**) A close-up view of a section, and a top view, of the moveable contact plate with the embedded pressure transducer and copper contact strip. All dimensions are in mm.

The flow facility, shown in detail in Figure 9a, utilized compressed air at 700 kPa that was regulated to 17 kPa by a Siemens 40 – 2 pressure regulator. The flow entered a Dwyer RMC 103-SSV flow meter that measured the mean flow rate, and further regulated the pressure. The flow then entered a 0.3m^3^ cylindrical plenum, representative of the lung volume. The inner walls of the plenum were insulated acoustically by foam. A 15cm long, 2.13cm^2^ cross-sectional area rectangular channel, representing the trachea, exited the chamber. The length of the channel was selected based on the results of numerical simulations of one-dimensional wave propagation that match the resonances of the subglottal pulmonary tract.

**Figure 10.**
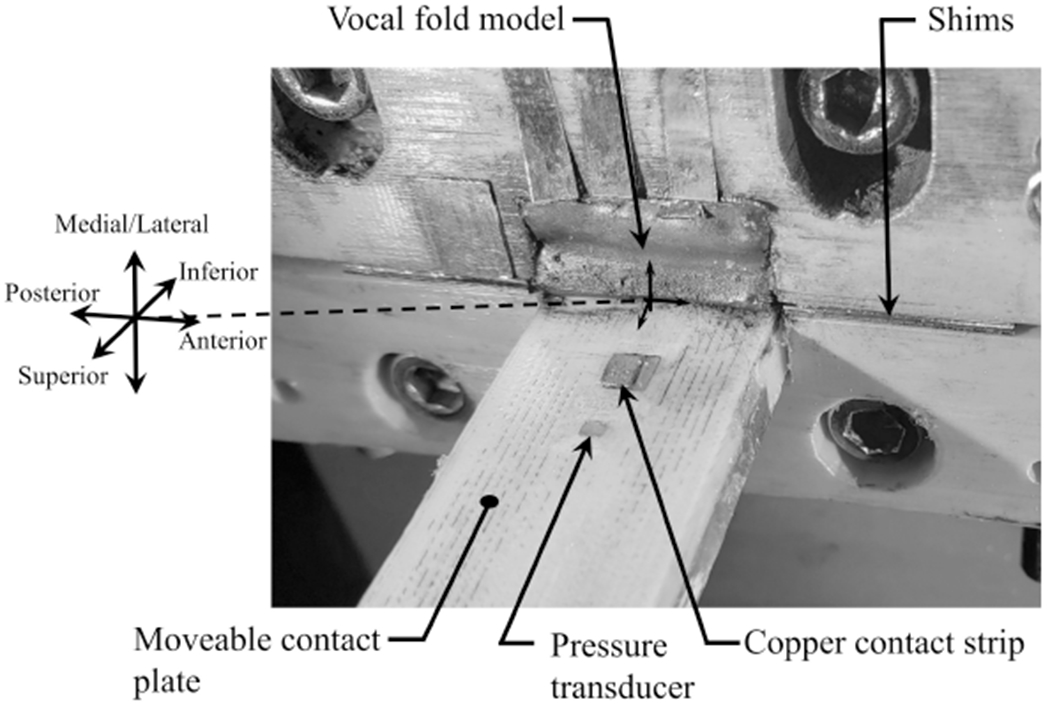
A close-up view of the hemi-vocal fold configuration, with the moveable contact plate. The location of the copper strip for measuring contact and the embedded pressure transducer are also noted.

Two opposing brackets that act as flanges were bolted to the end of the tracheal channel. Each bracket had a rectangular cut out in the middle; a synthetic VF model (see Section 3.1.1) was mounted in one, while the other allowed access for a moveable contact plate, against which the VF vibrated. The VF model mounted in the bracket is shown in Figure 10. The depth of the VF cut-out was 6.5mm, which resulted in the medial edge of the VF model protruding ~1.25mm above the top edge of the bracket. Shims were placed between the two opposing brackets to control the medial prephonatory compression (MPC) of the VF, which could be adjusted over a range of 0–1.25mm, and corresponded to the distance the model was deformed into the wall. The resultant static pressure at the corresponding MPC is referred to as the medial prephonatory pressure (MPP). At the inferior end of the bracket (not visible in Figure 10), the cut-out depth stepped up by 1.5mm. This ensured that the AT layer was embedded in the lateral wall, and that the inferior, angled surface of the VF model rose up from the lateral wall, as is shown schematically in Figure 9a.

A 17.0mm wide moveable contact plate was manufactured from polyactic acid (PLA) via rapid prototyping, and was placed in the channel of the opposing bracket (see Figures 9b and 10). It was centered under the VF model, creating a flat surface against which the VF vibrated. The plate extended the length of the tracheal channel. Superiorly, it extended past the exit plane of the VF model by ~10.0cm, where it was attached to a Thorlabs PT1 (Thorlabs, Newton, NJ, USA) linear slide that included a micropositioner with a Vernier scale. This allowed the inferior-superior position of the slide to be controlled with an accuracy of 0.025mm.

The unsteady subglottal pressure was measured with a Kulite ET-3DC (Kulite Semiconductor Products, Inc., Leonia, NJ) pressure transducer with a 6.35mm diameter sensing diaphragm that was flush-mounted in the wall of the tracheal channel, and positioned 3.0cm upstream of the VF entrance plane, as shown in Figure 9a. The subglottal pressure transducer has a FSO of 13.8 kPa with an accuracy of 0.5% FSO and a flat frequency response from DC to 2.5kHz. It was powered by an Omega PST-4130 power supply. A B&K 4189 (Brüell & Kær, Nærum, Denmark) 0.5 in (12.7mm) free field microphone with a dynamic range of 14.6–146dB over a frequency range of 6.3–20,000Hz was connected to a B&K 1704 power supply, and measured the radiated sound pressure level (SPL). The microphone was positioned obliquely at 45° to the exit of the VFs at a distance of 15cm. Both the subglottal pressure and radiated SPL signals were acquired with a National Instruments PCIe-6321 (National Instruments Corporation, Austin, TX, USA) data acquisition card, sampled at 80kHz. Data were acquired using a custom LabVIEW® program. High speed videos (HSV) of the VF kinematics were recorded with a Photron (Photron, Tokyo, Japan) AX200 camera oriented superior to the VF exit. The HSV was acquired at 20,000 frames/second, at a resolution of 640 pixels × 480 pixels. Light illumination was achieved with a 3500 lumen Ushio SugarCUBE Ultra (Ushio Inc., Tokyo, Japan) light-emitting diode (LED) light source. All data acquisition was synchronized by generating an external trigger using a custom-designed LabVIEW® program that simultaneously initiated acquisition across all measurement modalities. Finally, the entire experimental facility was located inside a Whisper Room SE2000 (WhisperRoom Inc., Knoxville, TN, USA) acoustic booth to mitigate ambient noise corrupting the SPL measurements, and to minimize acoustic reflections during data acquisition. The dimensions of the booth measure 187cm long by 126cm wide by 210cm high.

#### 3.1.3. Intraglottal and Vocal Fold Contact Pressure Sensor Integration

The findings from the contact pressure sensor validation measurements (see Section 2.3.2) were incorporated into the model hemi-laryngeal flow facility to enable accurate acquisition of intraglottal and VF contact pressure. A Millar Mikro-Cath™ pressure transducer was embedded in the moveable contact plate by 3D printing an enclosed interior channel with a 1.5mm wide (anteior-posterior)×1.3mm long (inferior-superior) opening window to the contact surface at the midplane of the contact plate (see Figure 9b). Note, the rectangular recess was slightly larger than the size of the sensing surface, but was smaller than the total pressure transducer size. The pressure transducer was threaded through the channel and positioned below the opening. The channel was then filled with Smooth-On® DS silicone, following the previously described approach (see Section 2.3.2) to ensure the top surface of the moveable contact plate is perfectly flat. It is worth noting that the inferior-superior length of the opening window that exposes the pressure sensor to the intraglottal and VF contact pressure was minimized to be 1.3mm. Initial attempts simply created a channel in the moveable plate that spanned the entire anterior-posterior width, for ease of manufacturing. However, while measuring VF contact with this configuration, high variability in the signal was observed, which was found to arise from slight superior-inferior motion in the VF models during vibration, which was most pronounced at the anterior-posterior midline. This resulted in the contact position of the VF varying spatially in the inferior-superior direction, along the anterior-posterior axis. Consequently, centering the pressure transducer in a channel spanning the entire anterior-posterior width of the plate resulted in the model VF contacting directly over the position of the pressure sensor at the anterior-posterior VF midpoint. However, at the anterior-posterior edges the channel was not in the zone of contact, and the pressure sensor was therefore exposed to the supraglottal pressure field. This occurred as the supraglottal pressure acted on the edges of the silicone channel, which transmitted a portion of the load onto the sensing surface of the pressure transducer via the deformable silicone. Consequently, significant bias and variability in the signal was observed. To address this, the anterior-posterior length of the sensing window was decreased to the current size of 1.5mm, to mitigate variations in contact length in the anterior-posterior direction. Sensitivity of the intraglottal and VF contact pressure to these subtle considerations are discussed in detail in Section 3.2.3.

#### 3.1.4. Identifying the Location of Vocal Fold Contact

In order to accurately measure the contact pressure during VF vibration, the location of contact must be known. Traditionally identified through visual observation [11,14], determining precisely where, or even if, contact occurs can be challenging. As will be shown in Section 3.2.2, small deviations in the location at which the contact pressure is measured can have significant impacts on the results.

**Figure 11.**
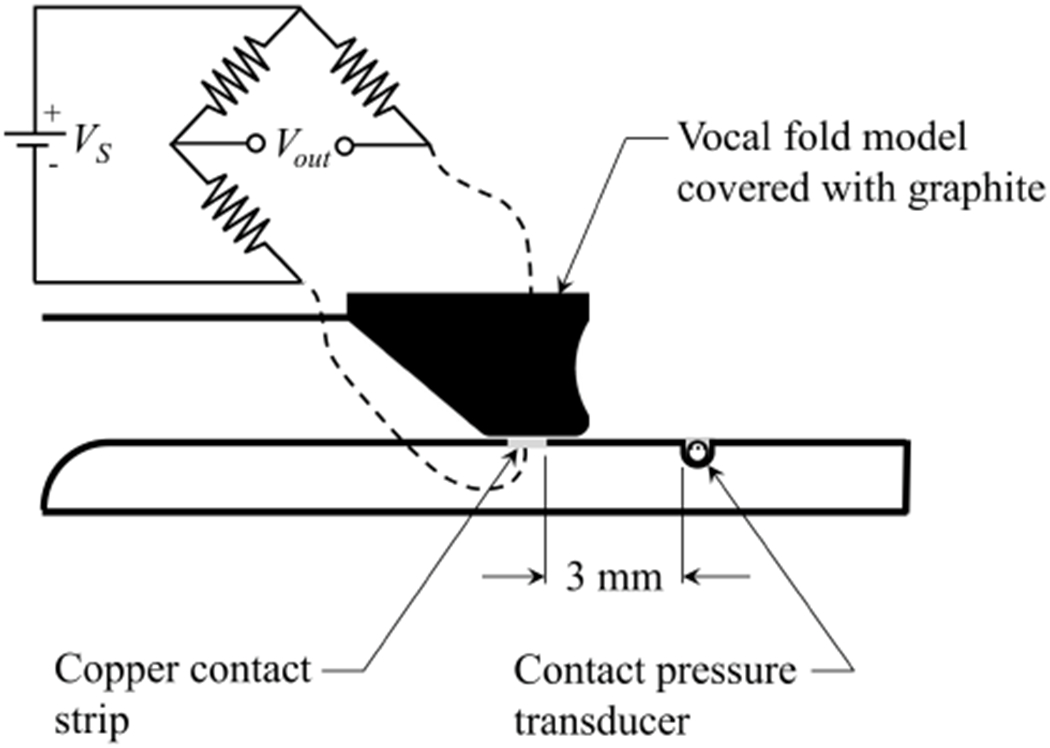
Schematic drawing of the hemi-vocal fold orientation and the Wheatstone Bridge circuit utilized for locating the point of contact.

In the current work, the contact location was determined using an approach first proposed by Syndergaard et al. [43], whereby a Wheatstone bridge was constructed, with one leg of the resistance arising from a circuit created by the VF and a conductive lead placed on the surface with which the VF impacts. A schematic of this approach is shown in Figure 11. The VF surface was coated with electrically-conducting graphite powder, while the other legs of the Wheatstone bridge were comprised of three, 330 kΩ resistors. A Kulite KSC-2 signal conditioner was used for generating the input voltage at 10V. The resistance through the VF leg was determined by monitoring the voltage output, *V*_out_. When the VF contacts the copper plate, it completes the circuit, and the resistance through that leg of the Wheatstone bridge decreases.

A 3mm wide piece of copper foil tape was embedded in the moveable contact plate by fabricating an interior channel with a rectangular opening on the face of the plate measuring 3.0mm long in the inferior-superior direction and 1.5mm wide in the anterior-posterior direction. The copper strip was inserted in the channel and exposed through the rectangular opening. The geometry of the slot was fabricated so that the exposed portion of the copper strip was flush with the surrounding surface of the plate. The orientation of the copper plate and its position relative to the pressure transducer is shown in Figures 9b and 10, respectively. The copper strip was positioned exactly 3.0mm inferiorly to the pressure transducer.

The inferior-superior location and length of VF contact was determined by first positioning the copper strip superior to the oscillating VFs. The micropositioner then moved the copper strip inferiorly, until the edge of the copper strip contacted the superior edge of the VF, which was identified by a sharp drop of 1–2 orders of magnitude in the resistance through the VF leg of the Wheatstone bridge. The position of the edge of the copper strip closest to the VF model was recorded, and the process was repeated with the copper strip starting out positioned inferiorly to the VF model, and the contact plate being moved superiorly until contact occurred. This procedure identified the inferior-superior length and position of contact of the VF model. Because the position of the copper strip on the plate is known relative to the pressure transducer, the pressure transducer could then be positioned at the midpoint of the inferior-superior contact length, or at any distance relative to that location.

### 3.2. Results

#### 3.2.1. Validation of Synthetic Vocal Fold Model Kinematics

**Figure 12.**
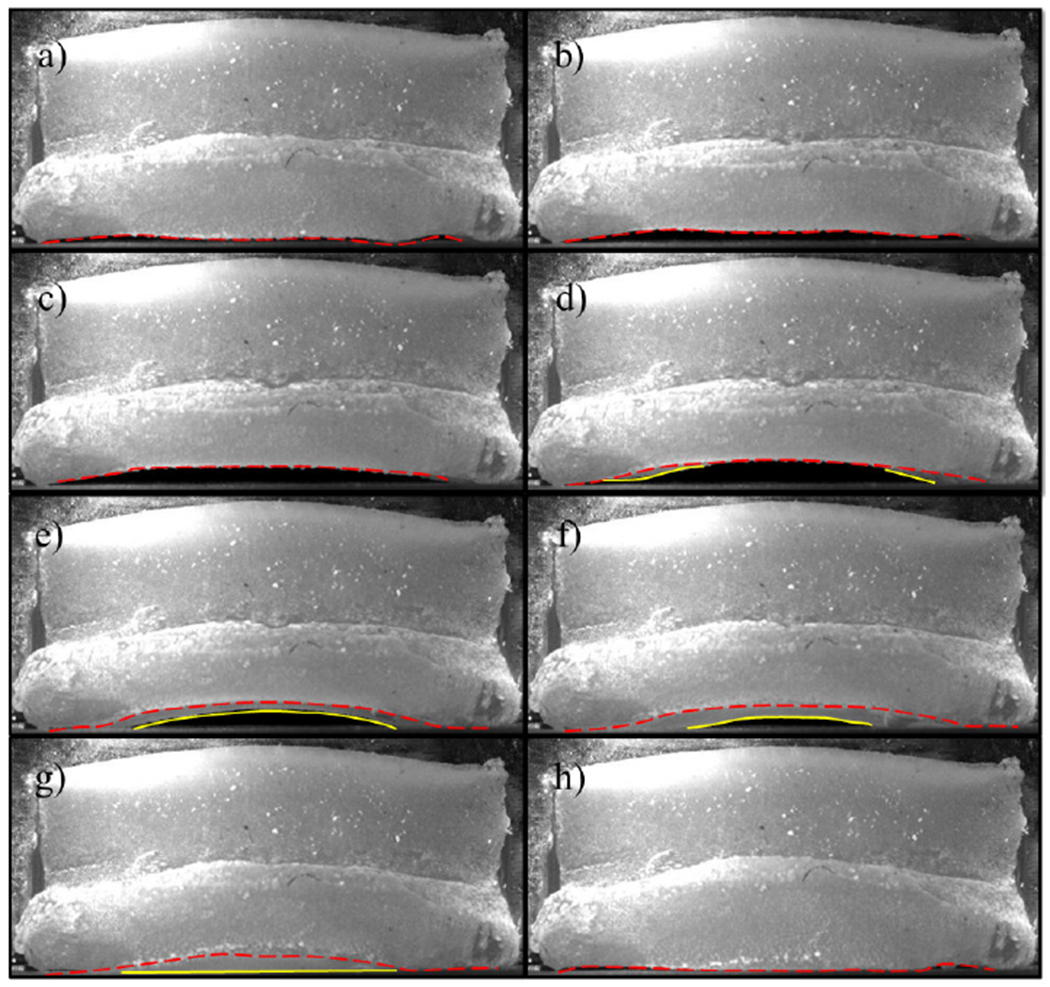
Extraction of eight instances in time, labeled sequentially as (**a–h**), throughout one cycle of the synthetic VF oscillation at a subglottal pressure of *p*_sub_ = 2.15 kPa (see also, Supplementary Video). The location of the superior (––) and inferior (–) VF edges are identified.

The kinematics of the hemi-VF arrangement were first investigated using the silicone VF model with a cover layer of *E_c_* = 0.95 kPa, which had an onset pressure of 1.6 kPa. The VF had a MPC = 0.75mm, which corresponded to MPP = 1.45 kPa. Eight images from HSV acquisitions were extracted at a subglottal pressure of *p*_sub_ = 2.15 kPa over the open phase of the glottal cycle, and are shown sequentially in Figure 12 (see also, Supplementary Material). The mean flow rate was *Q* = 409mL/s, and the fundamental frequency was *f_o_* = 168Hz. The primary point of excursion occurred about the midline, which is common in synthetic VF investigations with symmetric anterior-posterior boundary conditions [33]. A clear convergent-divergent motion is evident, as noted in Figure 12e–g, and is demarcated by the solid and dashed lines that indicate the inferior and superior VF margins, respectively.

**Figure 13.**
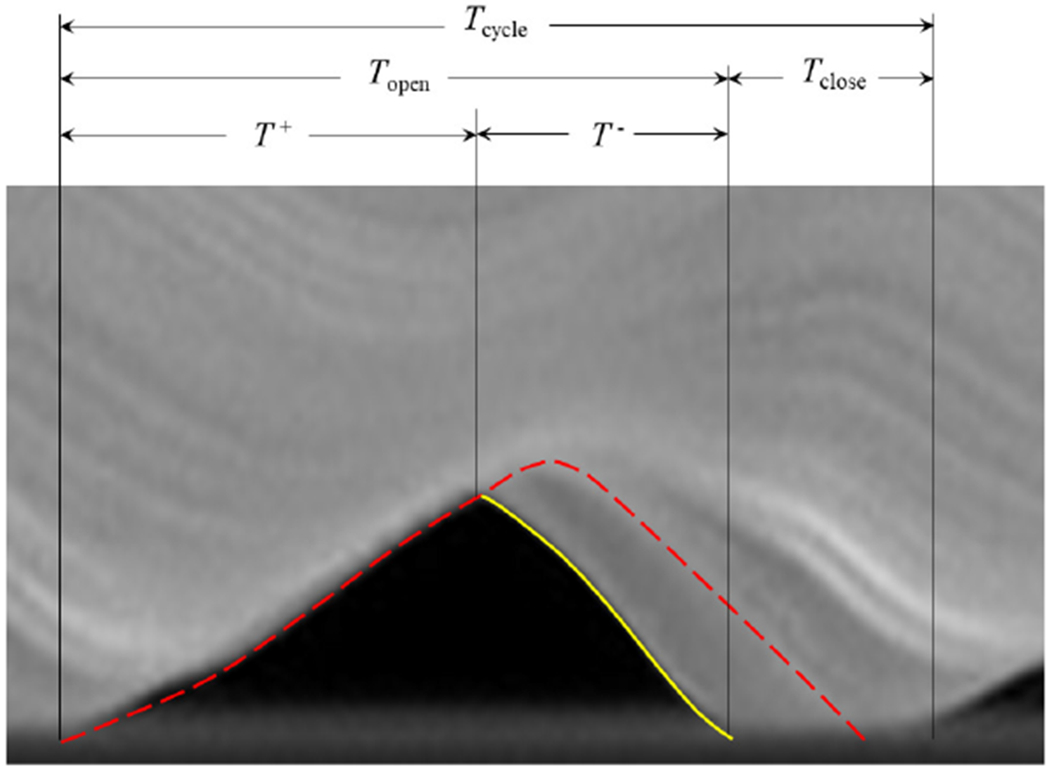
Kymogram plot from the HSV with the location of the superior (––) and inferior (–) VF edge identified.

A kymogram was extracted along the midline of the VF model, and is plotted in Figure 13 to highlight additional features of the oscillation. Again, a clear phase shift is identified between the superior and inferior VF edge, as denoted by the superimposed lines in the plot. The open quotient (*OQ*), defined as *OQ* = *T*_open_/*T*_cycle_ [44] was found to be *OQ* ≃ 0.74, where *T*_open_ and *T*_close_ are defined in Figure 13. This value is well within the physiological range that spans ~0.3–0.9, with a mean of ~0.6 [45–47], and is a significant advancement over prior modeling efforts that have reported values that are at the upper limit of the range of *OQ* [33,48]. In addition, the VF model presented herein produced a speed quotient, defined as *SQ* = *T*^+^/T^−^, of *SQ* ≃ 1.6; *T*^+^ and *T*^−^ are defined in Figure 13. Again, this is an important distinction of the current model, as prior synthetic modeling efforts typically find *SQ* values that are less than 1.0 [33], although physiological values range from 1.0 ≤ *SQ* ≤ 3.0 [46,49]. Finally, the maximum medial-lateral displacement was measured as 0.74mm, which is within the physiological range of reported values [50].

#### 3.2.2. Validation of Intraglottal and Vocal Fold Contact Pressure Measurements

Intraglottal pressure waveforms were acquired in the synthetic VF model with a cover layer of *E_c_* = 0.95 kPa, driven at a mean subglottal pressure of *p*_sub_ = 2.00 kPa. The mean flow rate was *Q* = 376mL/s and the fundamental frequncy was *f_o_* = 138Hz. The medial prephonatory compression was set to MPC = 0.75mm, which corresponds to a medial prephonatory pressure of MPP = 1.45 kPa. Following the procedure outlined in Section 3.1.4, the inferior-superior length of contact was measured to be 2.82mm. Note, this is longer than the size of the window opening on the moveable contact plate (1.3mm) in which the intraglottal pressure transducer was embedded. As such, when the intraglottal pressure transducer was placed at the midpoint of the contact location, the surface of the VF completely covered it during collision.

**Figure 14.**
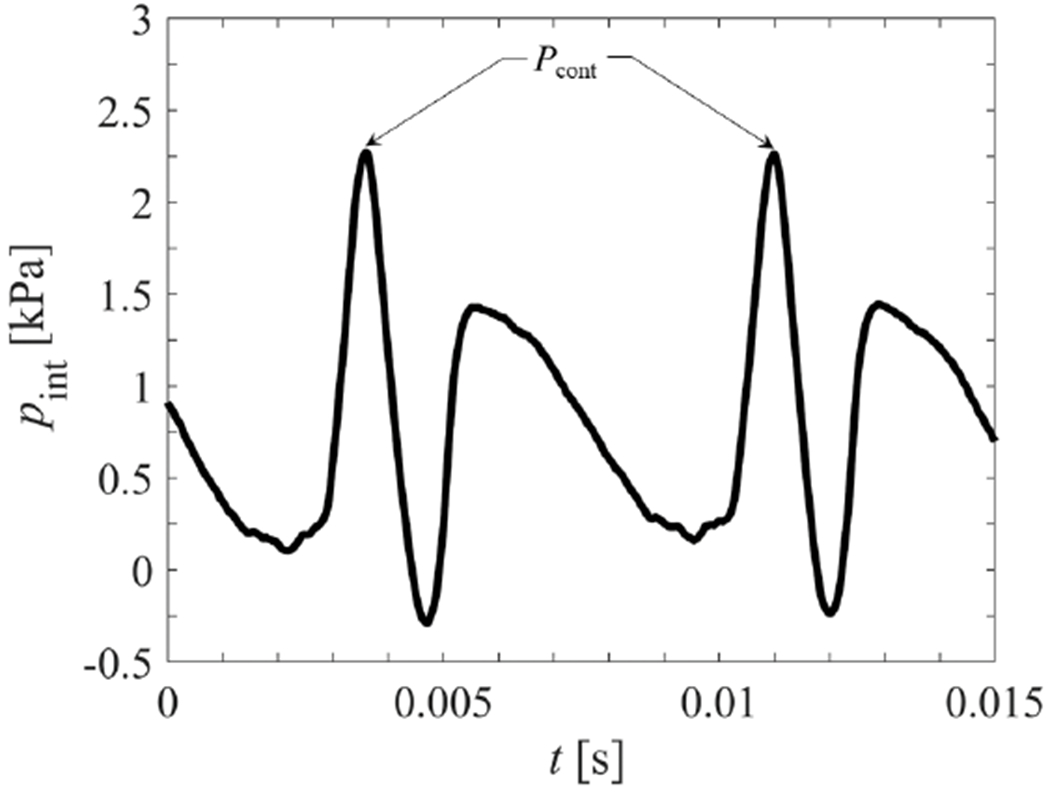
Intraglottal pressure waveform for *p*_sub_ = 2 kPa averaged over 100 cycles.

Figure 14 presents the intraglottal pressure waveform at the midpoint of contact, averaged over 100 cycles. Comparing the synchronized HSV with the intraglottal pressure measurements identifies that the first peak in the waveform corresponds to the maximum VF contact pressure, denoted as *p*_cont_ in Figure 14. The peak contact pressure is followed by a drop in pressure because of the equalization of internal stresses in the VF while the pressure sensor is still totally covered by the contact area. Progression of the mucosal wave results in the inferior edge of the VF surface lifting, and exposing the inferior edge of the transducer to the subglottal pressure while there is still complete closure. This produces the subsequent rise in the pressure, and the second peak. As the VF fully opens, the flow accelerates through the glottal opening, and the pressure decreases. This double peak pattern is analogous to the intraglottal pressure waveform acquired with canine VFs at the same subglottal pressure [11], as shown in Figure 1. In both cases, the measured contact pressure is higher than the mean subglottal pressure. The average contact pressure for the synthetic VF model, averaged over 100 cycles, is 2.29 kPa, which is 15% greater than the subglottal pressure. Prior excised experiments [11] reported a peak contact pressure of ~3 kPa with a subglottal pressure of 1.96 kPa. Nevertheless, very good agreement in the contact mechanics measured by the pressure sensor is observed. The difference in the ratio of *p*_cont_/*p*_sub_ between these two models may arise because of the different geometry and tissue properties of the excised VF and also variations in the medial prephonatory pressure, which was not specified for the canine investigations. In addition, the excised canine experiments [11] positioned the pressure transducer such that it protruded above the contacting wall by ~0.5mm, and physical protrusion of the sensor has been shown to overestimate the contact pressure (see Section 2.3.1).

#### 3.2.3. Sensitivity of Intraglottal and Vocal Fold Contact Pressures to Sensor Position

**Figure 15.**
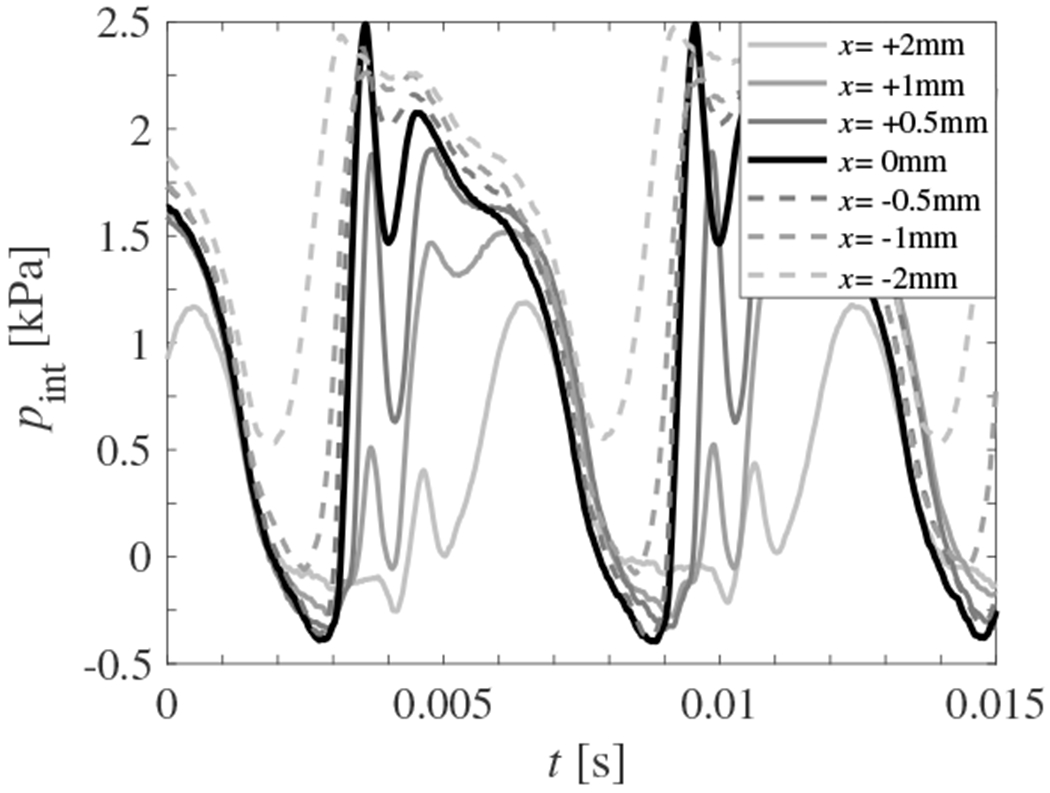
Intraglottal pressure waveforms as a function of pressure transducer position, acquired at *p*_sub_ = 2.15kPa. Superiorly increasing transducer locations are denoted by decreasing grayscale values for the solid lines, and a postive *x* position. Inferior positions are denoted by decreasing grayscale values for the dashed lines, and a negative *x* position.

Figure 15 presents 7 intraglottal pressure waveforms as a function of the inferior-superior position of the intraglottal pressure transducer, relative to the midpoint of the contact location, for the same measurements reported in Section 3.2.1 (*p_s_* = 2.15 kPa, *Q* = 409mL/s, *f_o_* = 168Hz, *E_c_* = 0.95 kPa, MPC = 0.75mm, and MPP = 1.45 kPa). Using the Wheatsone bridge arrangement, the inferior-superior contact length was measured to be 2.95mm. The coordinate system is defined such that *x* = 0 corresponds to the midpoint of VF contact with increasing positive values indicating the superior direction (denoted by solid lines of decreasing grayscale value), and negative values indicating the inferior direction (denoted by dashed lines of decreasing grayscale value). The contact pressure measurement locations that are plotted include *x* = 0mm, ±0.5mm, ±1.0mm, and ±2.0mm, measured from the inferior-superior midpoint of VF contact to the inferior-superior midpoint of the contact sensor. Remember that the sensing window placed in the moveable contact plate is 1.3mm long in the inferior-superior direction. Based on the length of contact in the inferior-superior direction, a portion or all of the pressure sensor will move outside the region of VF contact for locations at which *x* ≥ 0.825mm. That is, the sensor location of *x* = 0mm corresponds to the midpoint of contact, with the sensing window completely covered during contact. For the locations of *x* = ±0.5mm the sensing window is still completely covered during contact. When the sensor is positioned at *x* = ±1.0mm, the sensor is only partially covered during vocal fold contact, with part of it exposed to either the supraglottal, or subglottal pressure, respectively. Finally, for a position of *x* = ±2.0mm the sensor does not experience any contact, and is only measuring the supraglottal and subglottal pressure, respectively.

The maximum value of the contact pressure occurs when the intraglottal pressure transducer is centered about the midpoint of contact. Small adjustments in the position of the transducer (±0.5mm) lead to deviations in the peak contact pressure of up to 20%. Remember, these positions still correspond to the sensor being contained within the contact region.

As the pressure sensor is moved inferiorly from the midpoint of VF contact, the primary and secondary peaks merge, and the waveform begins to resemble the subglottal pressure signal. In particular, the minimum pressure value immediately following contact rises. This behavior can also be observed by comparing the waveforms at the midpoint of contact between Figure 15 and Figure 14, where only the subglottal pressure has changed. Note, the amount the pressure drops between the two peaks varies for these two scenarios. This arises due to very slight changes in the VF kinematics. Note that in Figure 14 the contact peak occurs at *t* ≃ 3.6ms. The secondary peak is reached when the inferior edge of the VF fold begins to open, exposing the sensor to the subglottal pressure, which occurs at *t* ≃ 5.5ms. In Figure 15 peak contact occurs at *t* ≃ 36 ms, but the secondary peak occurs at *t* ≃ 4.6ms; that is, the sensor is exposed to the subglottal pressure ~ 0.9 ms sooner. This results in the pressure signal not decreasing as much, and rising back to the secondary subglottal pressure peak sooner.

This same phenomenon is observed in Figure 15, where as the sensor moves inferiorly from the midpoint of contact, the minimum pressure that is reached in the valley between the contact and subglottal pressure peaks increases as the sensor is exposed to the subglottal pressure sooner, as seen in the case of *x* = −0.5mm. As the sensor is moved superiorly to *x* = +0.5mm it is exposed to the subglottal pressure later, which results in the valley between the VF contact and subglottal pressure reaching a lower value. In addition, as the sensor position moves inferiorly, it is moving into a region of increasing cross-sectional channel area below the glottis. In comparison to the pressure measurements at the midpoint of the glottis, when the VFs are open, the flow velocity (dynamic pressure) is lower. Consequently, the static pressure is higher.

As the sensor position is moved to *x* = ±1.0mm, the sensor measures the supraglottal/subglottal pressure simultaneously with the VF contact pressure, which biases the VF contact pressure higher/lower, respectively. For *x* = ±2.0mm only the supraglotta/subglottal pressure are recorded as the sensor is outside the region of contact. Note that at this position in the subglottal tract the subglottal pressure (second peak) does not reach as high of value as what is found at the midpoint of contact. This arises due to the acoustic resonances within the subglottal tract. When the probe is moved superiorly outside the contact region, the pressure similarly resembles the supraglottal acoustic pressure field. Interestingly, these findings also indicate that acoustic loading effects can have a significant impact on the dynamics of VF oscillation, as proposed by Titze (2008) [51].

## 4. Discussion and Future Work

### 4.1. Observations on Vocal Fold Model Development and Intraglottal and Vocal Fold Contact Pressure Measurements

The development of self-oscillating VF models in 2005 revolutionized scientific investigations of voiced speech production [29]. Since that time, significant advancements have been made, including: two- [30] and multi-layered models [32], the implementation of nonlinear material properties [52], geometries based on magnetic-resonance imaging (MRI) [33], and more recently, optimization of the layered geometry to improve mucosal wave propagation [53]. Nevertheless, progress is still needed to improve physiological relevance, particularly for investigations related to VF contact mechanics. The currently-employed VF geometry and structural layers addresses some of these shortcomings through the addition of a substrate layer of AT, and by modeling the curvature produced by the laryngeal ventricle on the superior surface of the VF. These modifications most notably decrease the open quotient and increase the speed quotient to physiologically-relevant values. These outcomes are a result of the robust mucosal wave that is observed during model oscillation, as captured via HSV observations. Although the subglottal pressure needed to drive the current VF models is slightly high, the hemi-VF arrangement is a contributing factor [11].

The suitability of the current VF geometry as a surrogate for clinical investigations of contact mechanics is justified by the ability to closely capture the key kinematics observed in excised canine [11] and human [54] VF investigations. Of particular importance in the current investigations is the ability to produce a sufficiently long inferior-superior contact length during collision. Initial investigations (not reported here) utilizing previously proposed geometries [32] resulted in excessively short inferior-superior contact lengths (~0.1 mm), where often, no contact could be measured in spite of visual inspection by HSV suggested it occurred. The length of contact in the inferior-superior direction also becomes an important consideration when attempting to measure collision because for sensor sizes larger than the inferior-superior contact length, the recorded pressure will be a spatial average of both the contact pressure, and the subglottal and/or supraglottal pressure, thereby biasing the results. This can produce drastic variations in measures of peak contact pressure. In addition, the need to maintain a flush contact surface that transmits the loading directly onto the sensing element of any transducer is an important consideration that impacts the fidelity of the measurements because geometric disturbances arising from probe interference can greatly influence the accuracy of contact pressure measurements, as discussed in Section 2.

The dependence of the peak contact pressure on the inferior-superior position of the intraglottal pressure transducer and, more importantly, its sensitivity to probe placement, was an important finding. While important to consider during experimental investigations, it is of utmost relevance in clinical investigations, where precise control of contact pressure sensor location will depend on variables such as laryngeal anatomy, tolerance of the subject for invasive endoscopic procedures, and the skill of the clinician/surgeon. Most notable was the observation that the peak contact pressure varied a relatively large amount (up to 20%) as a function of inferior-superior sensor position, even when the sensor was completely covered by the VFs during contact. When the sensor was positioned such that it was only partially covered during VF contact, the peak pressure changed drastically. Due to the variation in both the magnitude of the peak contact pressure, and the timing between the contact and subglottal pressure peaks, care should be taken when interpreting intraglottal pressure signals as they are highly dependent upon the size of the pressure sensor relative to the inferior-superior length of VF contact.

### 4.2. Recommendations for In Vivo Assessment of Sensor Location

Nevertheless, there are features within the intraglottal pressure signal that can guide an investigator and help identify when the location of peak contact pressure has been located. Notably, a double-peak should be present in the signal, with the magnitude of the primary contact peak on the order of the subglottal pressure, although VF posturing and medial compression can lead to variations in these ratios. Instructions given to a participant to phonate using pressed versus breathy voice at the same vocal effort during clinical investigations can exploit this dependency by correspondingly amplifying or decreasing the primary contact peak, respectively, while not affecting the magnitude of the secondary peak. This approach may aid in identifying the primary contact peak and thereby aid in locating sensor placement relative to the peak contact pressure location. Additional strategies to aid in sensor placement during clinical examination have been devised, such as placing two sensors on a probe, separated in the inferior-superior direction, to identify both the subglottal and contact pressure simultaneously, thereby guiding probe location through a comparative approach between the two signals [54].

Probe size relative to inferior-superior contact length should also be carefully considered. While a smaller probe is advantageous in that it provides a higher spatial resolution of the VF contact pressure, positioning becomes increasingly difficult. Contrarily, it is imperative that any pressure probe utilize a dimension in the inferior-superior length that is smaller than the inferior-superior contact length, to avoid biasing the signal by simultaneously measuring the VF contact pressure and the subglottal and/or supraglottal pressure.

These findings highlight the importance of: (1) utilizing an independent measure to verify contact that is not dependent upon visual observation; (2) designing intraglottal pressure sensors/approaches to prevent disruption of contact mechanics due to intrusion of the physical probe; and (3) considering how the size and positioning of a contact sensor relative to inferior-superior VF contact length may influence the measurement and interpretation of contact pressure results.

Finally, an additional concern that exists with the Millar Mikro-Cath™, but that has not been addressed up to this point, is the sensitivity of the pressure transducer to temperature variations. The benchtop nature of the current investigations facilitated maintaining the transducer at a constant temperature so as to mitigate this problem. The pressure transducer has a quoted temperature sensitivity of ±0.27 kPa for a temperature variation from 25–40 °C. Care should therefore be taken when implementing it in *in vivo* investigations where thermal shifts may arise due to radiative heat transfer from the lighting source to the transducer, as well as convective heating/cooling due to the warm glottal environment, and the airflow that is passing over the transducer. A strategy for addressing this concern is to zero the signal from the sensor while it is immersed in warm water that is equal to the anticipated/measured temperature of the air passing through the glottis. If this is performed immediately prior to any measurements, it will minimize uncertainties arising from thermal drift.

## 5. Conclusions

The accuracy and sensitivity of acquiring intraglottal and VF contact pressure acquisitions has been investigated using a custom facility that enables validation with ground-truth pressure measurements, and a physical hemi-laryngeal flow facility. To facilitate physiologically-relevant investigations of VF contact, a new description of the geometry for synthetic, self-oscillating VF model geometry was introduced. The VF model exhibited a robust mucosal wave resulting in more realistic kinematic measures of open quotient, speed quotient, and inferior-superior contact length, when compared to previously proposed synthetic VF model geometries.

The impact of sensor intrusion on contact mechanics was quantified, with very small disturbances (O(1mm)) causing significant deviations in the measured contact pressure. To mitigate these effects, a method for embedding the pressure transducer within the contacting plate and encapsulating it within silicone to create a flat contact surface was introduced and validated. This approach was then applied to measures of intraglottal and VF contact pressure measurements in a synthetic VF model. Features of the intraglottal pressure waveform were identified, and their dependency on small variations (O(0.5mm)) in sensor position relative to contact location were highlighted.

These findings and insights are applicable to *in vivo* intraglottal pressure probe designs, as well as strategies that can be implemented by the investigator performing the *in vivo* investigations to accurately identify when the desired, subglottal, intraglottal, or supraglottal pressure is being acquired.

## Supplementary Material

Video 1

## Figures and Tables

**Table 1.** Vocal fold model and physiological values of the moduli of elasticity for the different layers of the vocal folds. Smooth-On® Ecoflex 0030 and Dragonskin Silicone mixtures are denoted by (EF) and (DS), respectively.

Layer	Physiological Range (kPa)	Vocal Fold Model [Std. Dev.] (kPa)	Silicone Ratio (Type of Silicone)
Adipose tissue	1 – 10 [39]	6.8[0.0058]	1 : 1 : 4 (EF)
Body	1.5 – 50 [35,41,42]	17.3[0.042]	1 : 1 : 2 (EF)
Cover	1 – 8 [35,36]	0.95[0.053]	1 : 1 : 8 (EF)
Epithelium	Not available	101[1.4]	1 : 1 : 1 (DS)

## Featured Application:

A new method for measuring intraglottal and vocal fold contact pressure is presented, and the accuracy and sensitivity of the approach is discussed. The outcomes are applicable to efforts that aim to directly measure *in vivo* intraglottal and vocal fold contact pressure during phonation.

## Abbreviations

The following abbreviations are used in this manuscript:

AT: Adipose tissue
DS: Smooth-On® Dragonskin 10
EF: Smooth-On® Ecoflex 0030
FSO: Full scale output
HSV: High-speed video
MPC: Medial prephonatory compression
MPP: Medial prephonatory pressure
*OQ*: Open quotient
PGO: Posterior glottal opening
SPL: Sound pressure level
*SQ*: Speed quotient
VF: Vocal fold
